# The ethical dimensions of wildlife disease management in an evolutionary context

**DOI:** 10.1111/eva.12171

**Published:** 2014-06-24

**Authors:** GKD Crozier, Albrecht I Schulte-Hostedde

**Affiliations:** 1 Canada Research Chair in Environment, Culture and Values, Department of Philosophy, Laurentian UniversitySudbury, ON, Canada; 2 Canada Research Chair in Applied Evolutionary Ecology, Department of Biology, Laurentian UniversitySudbury, ON, Canada

**Keywords:** conservation, culling, decision tree, vaccination, values.

## Abstract

Best practices in wildlife disease management require robust evolutionary ecological research (EER). This means not only basing management decisions on evolutionarily sound reasoning, but also conducting management in a way that actively contributes to the on-going development of that research. Because good management requires good science, and good science is ‘good’ science (i.e., effective science is often science conducted ethically), good management therefore also requires practices that accord with sound ethical reasoning. To that end, we propose a two-part framework to assist decision makers to identify ethical pitfalls of wildlife disease management. The first part consists of six values – freedom, fairness, well-being, replacement, reduction, and refinement; these values, developed for the ethical evaluation of EER practices, are also well suited for evaluating the ethics of wildlife disease management. The second part consists of a decision tree to help identify the ethically salient dimensions of wildlife disease management and to guide managers toward ethically responsible practices in complex situations. While ethical reasoning cannot be used to deduce from first principles what practices should be undertaken in every given set of circumstances, it can establish parameters that bound what sorts of practices will be acceptable or unacceptable in certain types of scenarios.

## Introduction

The application of evolutionary principles to medicine, agriculture, and conservation is widespread (Ashley et al. 2003; Hendry et al. 2011), but the ethical implications associated with such applications are rarely considered. The inter-relatedness of human populations, domestic animals, and the natural environment explicitly including wildlife is reflected in paradigms such as the One Health Initiative, which calls for the integration of various silos including the medical, veterinarian, and ecological science communities to respond to growing zoonotic threats (Dahal and Kahn 2014). In this context, sound wildlife disease management requires careful attention to applied evolutionary ecology and to ethical conservation because of the implications for conservation and public health.

For example, some contemporary diseases affecting wildlife populations are a direct or indirect consequence of human activities, including anthropogenic climate changes that modify the traditional ranges of wildlife and pathogens alike, and the fragmentation of wildlife habitats that leads to denser wildlife populations, which then present ideal conditions for the transmission of pathogens (Suzán et al. 2012).^1^ Furthermore, disease management practices risk causing more harm than good if they are not designed with careful attention to the potential for evolutionary and ecological mechanisms that can lead to additional negative outcomes. For example, some commonly used management practices can increase disease virulence by intensifying adaptive pressures on pathogens, or they can detrimentally affect other species in the ecological community (McCallum 2009). Additionally, wildlife disease management practices ought to contribute to evolutionary ecological research (EER) regarding wildlife diseases, because this provides a feedback mechanism critical for the on-going improvement of management practices (i.e., adaptive management). Explicitly, an adaptive management approach to wildlife disease precludes practices that are not only politically motivated but wherein the political objectives are deliberately pursued in such a way that they are not informed, or are even contraindicated, by EER; such measures are unethical, in part, because they are motivated by short-term, partisan gains rather than being directed toward the long-term overall reduction of harm.

Herein, we propose a two-part framework to assist decision makers in identifying (and thereby avoiding) the potential ethical pitfalls of wildlife disease management. The first part consists of six values – freedom, fairness, well-being, replacement, reduction, and refinement. These values have been developed for the ethical evaluation of EER practices, and this study shows that they are also suited for evaluating the ethical dimensions of wildlife disease management. The second part consists of a decision tree to assist decision makers identify the ethically salient dimensions of wildlife disease management and guide them toward ethically responsible practices in complex situations. Although ethical reasoning cannot be used to deduce from first principles what practices should be undertaken in all circumstances, it can establish parameters that bound what sorts of practices are acceptable or unacceptable in certain types of scenarios. When both science and ethics agree on such measures, it exemplifies how good science can be ‘good’ science – that is, how effective science is also science that is conducted ethically.

To begin, we define specific terms for the purposes of our argument. When we discuss the ethics of wildlife disease management, by ‘ethics’ we refer to a subset of the norms prescribing acceptable behavior in human social groups that is taken to be more fixed and fundamental than legal structures or rules of etiquette.^2^ Several recommendations emerge as particularly appropriate with respect to ethics and applied evolution. Specifically, both theoretical constructs indicate that measures ought to be taken to maximize the benefits of any wildlife disease management program for the most vulnerable parties – whether human or nonhuman animal. By ‘vulnerable parties,’ we refer to any animals – human or not – that suffer negative effects from the management (or nonmanagement) of wildlife diseases. We distinguish ‘vulnerable parties’ from ‘vulnerable populations,’ using the latter term to refer more specifically to populations that are susceptible to infection from the wildlife diseases in question. Thus, all members of a ‘vulnerable population’ are arguably ‘vulnerable parties,’ but some ‘vulnerable parties’ will not be members of ‘vulnerable populations’.

## The case of bovine tuberculosis in Eurasian badger populations

The recent case of bovine tuberculosis (*Mycobacterum bovis*) and the culling of Eurasian badger (*Meles meles*) underline the need for integrating sound science with the consideration of ethical perspectives in wildlife disease management. The Eurasian badger has been linked with the spread of tuberculosis to cattle, leading to significant economic losses (Gross 2013). Vaccination of cattle against tuberculosis is not yet perfected (Buddle et al. 2011), and it is explicitly forbidden in legislation by the European Union because current vaccine practices in cattle may not sufficiently protect against bovine tuberculosis (EFSA 2013). As it stands, vaccinated cattle may become infected by *M. bovis* and spread the disease and would be indistinguishable from noninfected vaccinated animals (EFSA 2013). Vaccination of badgers is effective and leads to a significant reduction in the severity and progression of bovine tuberculosis (Carter et al. 2012) but is considered too expensive (Gross 2013). Culling of badgers has been undertaken, with controversy surrounding its efficacy (Pope et al. 2007). Some have concluded that if a cull were to be effective, it would have to be over a very large area, making the use of a cull impractical and costly (Macdonald et al. 2006). Complicating this situation is the value that Eurasian badgers hold in the United Kingdom, in part, because of its role in children’s literature, with animal lovers protesting the killing of members of this species. While the epidemiology of the disease is complex, and therefore, the best practices of disease management unclear (Hofer 2013), the need for ethical considerations is evident in terms of mitigating animal welfare issues and designing a management program that takes into account ethical values.

## Reflections on current practices

Although there are alternatives involving isolation and quarantine, or, increasingly, vaccination of the reservoir population (the population of hosts that harbors the pathogen), culling is the primary short-term control technique in wildlife disease management. It is also the most ethically problematic (Littin and Mellor 2005). Poisons, hunting bounties to encourage shooting and trapping, electrified fences, and even deadly pathogens have been used to reduce infected wild populations (Wobeser 2002; Littin and Mellor 2005; McCallum 2009).

These approaches to disease management are based on the notion that disease transmission is density-dependent, and thus by reducing the sizes of reservoir populations, there are fewer susceptible animals available for the pathogen to infect, and, consequently, contact between infected and uninfected animals is reduced, thereby decreasing the frequency by which the pathogen transmits to naïve hosts (McCallum 2009; Smith et al. 2009). While this approach underlines human epidemiology, natural populations of wildlife and their pathogens do not necessarily behave in a density-dependent manner (e.g., Cross et al. 2013b), leading to poor results from these kinds of management programs in some circumstances (Lloyd-Smith et al. 2005). The use of culling as a method in controlling zoonoses explains why the efficacy of this technique is not certain and is often dependent on the behavior and ecology of the host species (Woodroffe et al. 2006; Johansen and Penrith 2009). The use of culling can also have political motivations that are not consistent with scientific evidence (Torgerson and Torgerson 2010; Spencer 2011).

Animal welfare is a significant concern when culling methods are involved in wildlife disease management. Killing a sentient animal for purely instrumental purposes (i.e., when there is no benefit to the animal itself, unlike when suffering animals are euthanized) should never be undertaken lightly. To do so would be to erroneously assume that these animals have no intrinsic value as individual, sentient beings, despite the fact that many of them have complex cognitive, affective, and social characteristics (Vucetich and Nelson 2007). It is not a simple exercise to determine when culls of reservoir populations are ethically warranted; however, it is possible to articulate the salient considerations. For example, a utilitarian calculus can serve as a means for appealing to rational consistency in decision making (Vucetich and Nelson 2007). This can be constructed such that the costs (in terms of loss of life) to the individuals being killed are weighed against the benefits to be gained from the killing (such as the protection of vulnerable populations or habitats). As long as the individual animals’ intrinsic interests are included in this calculus, (arguably, though not uncontroversially) they are not merely being treated as a means to an end.

Although such calculations are rarely value-neutral, because a variety of rationales can be drawn upon to assign different values to the lives of individuals and to the benefits and costs to populations, some rationales are better than others. For example, a rationale that assigned the life of nonhuman animals equal weight to the life of humans might be inappropriate in the context of wildlife disease management. First, pragmatically speaking, it would set the bar too high; it is not very controversial that it would be unethical to cull human populations to stop the transmission of infectious diseases, but applying these same standards to nonhuman animals would bring wildlife disease management as a field to a virtual halt. Second, from a philosophical standpoint, such a rationale would fail to recognize that humans have special ethical rules that apply to humans specifically because we are conspecifics engaged in particular ‘social contracts’ with one another. This does not mean that we could not opt to include nonhumans in similar rules; however, due to our social and evolutionary history, certain ethical conventions currently apply specifically to our interactions with other humans. Third, this rationale is not sufficiently sensitive to the importance of evolutionary and ecological considerations – of the relationship between these individuals and the evolving populations of which they are a part and in which they share some value.

A preferable rationale is grounded in the connection between animal welfare and conservation. Whereas welfarists focus on the well-being of individual animals, conservationists are largely concerned with populations and habitats; however, where they share common ground is in the integral connection between the welfare of individual animals and the integrity of the populations of which they are members and the ecosystems that they inhabit (Paquet and Darimont 2010; Wall 2010). A commitment to both animal welfare and conservation are distinct from a commitment to animal rights, insofar these former commitments are consistent with killing sentient animals under certain circumstances. On this rationale, a utilitarian evaluation of the costs and benefits of a cull would have to recognize that individual animals also share an interest in the ecological communities of which they are a part. Thus, for example, if the harm caused by an exotic or anthropogenically accelerated pathogen can only be mitigated by a controlled cull of a reservoir population, with the goal of stabilizing the habitat in which that population can then thrive as it had previously, then the individuals culled could be said to share an interest in this outcome. This consideration would need to be included in the utility calculus; this ‘inclusive interest’ is one way in which the death of an individual animal to benefit others need not be purely ‘instrumental’.^3^

This is not to advocate for therapeutic interventions on behalf of wild populations or individual infected wild animals, but rather to emphasize that, from an ethical perspective, the threshold for lethal intervention is lower when the reservoir population is itself suffering from the effects of the disease. Admittedly, it sounds odd to discuss intervening in ‘natural ecosystems’ to rescue populations from ‘natural diseases’ – and for these reasons wildlife managers have preferred to adopt a ‘hands-off’ approach in most cases, excepting when endangered species are threatened. It must be borne in mind, though, that many emerging wildlife diseases are not part of the evolutionary history of these wildlife populations but are new threats that have developed because of anthropogenic environmental change; EER and disease management must take into account whether diseases are exotic, emerging, or endemic, and adjust strategies accordingly. As such, adopting a uniform ‘hands-off’ approach to epidemics devastating wildlife populations to allow ‘nature to take its course’ could be somewhat disingenuous. For example, European tourists likely introduced white-nose syndrome – a fungal infection (caused by *Pseudogymnoascus destructans*) that is threatening many species of North American bats (Warnecke et al. 2012; Cryan et al. 2013). Letting these species go extinct from an exotic pathogen introduced by humans would be inconsistent with the ethical obligation to conserve biodiversity.

Additional concerns are raised when the reservoir population itself suffers little or no direct harm from the disease. In such cases, when can culling wild disease carriers be justified by the benefit this offers to the vulnerable population that the cull is designed to protect? When the disease is zoonotic, the vulnerable population is one to which we have special duties – given the strong, widely recognized, and well-defined responsibilities humans have to protect members of our own species, it can be relatively easy to justify culling of wild reservoir populations in such cases. But what if the vulnerable population is an endangered species; or what if it is valuable domestic livestock, and the main motivation for culling wild reservoir population is economic, such as Wyoming cattle threatened by brucellosis (caused by *Brucella abortus*) carried by wild bison (*Bison bison*) and elk (*Cervus elaphus*) populations (Bienen and Tabor 2006; Cross et al. 2013a)? What if the vulnerable population is valued primarily for sport hunting, such as when chronic wasting disease affects deer populations (Cullingham et al. 2011)? And how would the calculation change if the hunting were for subsistence instead of sport, as in toxoplasmosis (*Toxoplasma gondii*) affecting beluga whales (*Delphinapterus leucas*) – a primary source of nutrition for Inuit communities (Grigg et al. 2014; University of British Columbia 2014)?

Less invasive than culling, vaccination has proven a viable solution in some cases, such as rabies treatment using baited food (Rosatte et al. 1992; Nunan et al. 2002). This can be conducted noninvasively by feeding the wild populations foods containing the vaccine and as such offers significant benefits in terms of animal welfare over culling methods. However, the practice of vaccinating wild populations is fraught with complications, including problems in developing vaccines that can be effectively distributed to the target reservoir population (herbivores, e.g., are much more difficult to lure to bait stations), prohibitive costs associated with vaccinating populations at a broad spatial scale, the presence of multiple hosts that may require unique vaccines, and management of a vaccine program that requires organizational cooperation across multiple jurisdictions (Cross et al. 2013a). Furthermore, vaccination programs are not without their own risks, which we discuss in the following section.

Other less harmful methods of wildlife disease management have been employed, with varying degrees of success. Individual treatment of diseased wildlife might seem to be the most humane option insofar as the animals in the reservoir populations are healed where possible and only killed when necessary to ease the individual’s suffering. Consequently, conservationists favor this management technique; however, it is not practical or effective in larger populations. It also is not harm-free, as handling and release of animals can permanently damage group social dynamics, and also bring danger of mortality, for example through stress-induced suppression of the animals’ immune systems (Burrows 1992; Cattet et al. 2008).

Population dispersion control methods have also been introduced, such as setting up wildlife feeding stations or introducing disruptive stimuli to induce or provoke wildlife to move away from populations vulnerable to transmission. As with culling, however, these methods have often been applied without due attention to the ecological and evolutionary context. For example, establishing feed stations for elk increased the likelihood that elk would encounter aborted fetuses, leading to an increase in the transmission of brucellosis (Bienen and Tabor 2006; Creech et al. 2012; Cross et al. 2013a).

## Being mindful of evolutionary ecology

Any management strategy employed to mitigate the effects of wildlife disease should be mindful of potential evolutionary consequences. Indeed, most strategies associated with wildlife disease management, particularly of zoonotic pathogens, result in short-term results that are effective at an ‘ecological’ time scale; yet, these same strategies can have profound consequences for host-pathogen coevolution, including enhanced or reduced virulence, and changes in transmission dynamics (Gandon and Day 2007).

From a pathogen’s perspective, removing potential hosts from a population can lead to the evolution of enhanced virulence under certain conditions (Mackinnon et al. 2008). Culling, for example, can increase selection pressure on the pathogen, effectively enhancing its capacity to cause disease (Shim and Galvani 2009). It can also cause the disease to spillover into domestic livestock populations when the supply of wild hosts is reduced and with increased dispersal of animals in the reservoir population due to disruption of social groups (Carter et al. 2007). Vaccination strategies can also lead to evolution of enhanced or reduced virulence, depending on the mode of transmission and other ecological factors (Day et al. 2008). Vaccines typically must also be distributed to a sufficient proportion of the population in order to develop the ‘herd immunity’ required to minimize transmission of the pathogen, and failure to vaccinate a sufficient percentage of the population can increase adaptive pressures on the pathogen (Lloyd-Smith et al. 2005).

Additionally, vaccination can itself pose a risk to wild populations, including morbidity and mortality due to vaccine reactogenicity (Paoletti 1996; McCallum 2009). Care must also be undertaken to avoid critically undermining populations with a vital ecological role, such as top carnivores or pollinators, because species reintroduction is extremely challenging or impossible once a local population has been extirpated. Trophic cascades from such losses can have profound effects on the evolutionary pressures acting within an ecosystem.

Decision makers often poorly understand these evolutionary considerations; and additionally, there is an impetus to overlook them when short-term, (often politically motivated) crisis management is the primary objective, rather than long-term evolutionary sustainability. For example, the desire to remove bovine tuberculosis and brucellosis (*Brucella abortus*) from a population of plains bison (*Bison bison bison*) from Wood Buffalo National Park in Canada involves various measures including extensive medication and culling that may minimize the risks of disease transmission, yet little consideration is made as to the consequences of these efforts on pathogen evolution (Nishi et al. 2002).

A long-term, evolutionary perspective that prevented disease by redistributing resources to prevent transmission or increasing landscape connectivity to reduce population densities would not only minimize the negative evolutionary consequences that some short-term practices impose, but would also have a positive effect on individual animal welfare because of the limitation of practices such as culling. Additionally, consideration must be given to the role played by the pathogen within the ecosystem; an endemic disease might play an important role in the evolutionary history of the reservoir population, and eliminating it for the purpose of protecting recent developments in human agriculture could risk destabilizing the system. Serious consideration must be given to finding management strategies that support commitments to both animal welfare and conservation, which might require scaling back human incursions into the habitats of reservoir populations and pathogens. Of course, such measures can be costly to local human communities (who might need to be adequately compensated), but the long-term evolutionary price incurred by ignoring these considerations for EER could be much higher.

One consideration that has not received sufficient attention is that a conservation mandate to protect biodiversity might imply the need to protect pathogens from extinction in part because of their ecological role as consumers. Insofar as wildlife disease eradication programs are aimed at minimizing or extirpating the organism directly responsible for mortality and morbidity in wildlife, livestock, and human populations, they might undermine biodiversity conservation. In other words – does conservation of biodiversity extend to disease-causing organisms, particularly if these organisms are rare and phylogenetically isolated? For instance, in 2011, rinderpest – a disease affecting cattle and buffalo, and often referred to as the ‘cattle plague’ – became the second disease-causing virus (after smallpox) to be officially eradicated in the wild through a deliberate global campaign; yet minimal samples of the virus remain stored in high containment facilities for research purposes. As in the case of the variola virus, responsible for smallpox, there is vigorous on-going debate regarding whether conserving samples of wildlife viruses *ex situ* is justified (whether for research purposes or whether it is someday needed to synthesize a vaccine in case it re-emerges in natural populations from an unexpected source) given the risk it poses for reintroduction into the wild (Voyles et al. 2009). Additional consideration should be given, however, to the information contained within the pathogen’s genome and the evolutionary history it represents because it might be of value (Redding et al. 2010). For example, phylogenetic isolation has been used as a unique value for prioritizing species conservation efforts (Redding et al. 2010). If conservation of biodiversity extends to pathogens (Whiteman and Parker 2005; Nichols and Gomez 2011; Gomez and Nichols 2013), then a disease-causing organism that is in some way determined to be evolutionarily ‘unique’ can be objectively argued to have conservation value.

## Six values provide the conceptual & linguistic framework for ethical analysis

We propose a two-part framework to assist decision makers identify ethical pitfalls of wildlife disease management. The first part consists of six core values: freedom, fairness, wellbeing, replacement, reduction, and refinement (Table 1). We have advocated elsewhere (Crozier and Schulte-Hostedde 2014) that these six values are well suited for evaluating the ethical dimensions of EER, in general (i.e., beyond the more limited domain of ecologists specializing in wildlife disease management), and we argue here that they are also ideally suited for evaluating the ethical dimensions of wildlife management. The second part presents a decision tree to help identify the ethically salient dimensions of wildlife disease management and guide managers toward ethically responsible practices in complex situations.

**Table 1:** Six core values drawn from principles of bioethics (Beauchamp and Childress 1977) and animal welfare (Russell and Burch 1959) and their application to ecological research and wildlife disease management.

Values	Original Application	Application to Ecological Research	Application to Wildlife Disease Management
Freedom	Respect the rights of people and their communities to make decisions for themselves	Avoid jeopardizing locally valuable resources without consultation with, and consent from, local communities	Avoid jeopardizing locally valuable resources without consultation with, and consent from, local communities
Fairness	Treat people and communities with respect for justice	Balance the interests of various parties affected by the research	Balance the interests of various parties affected by the management practices
Wellbeing	Seek to maximize the health and happiness of individuals and their communities, and do not act to undermine their health and happiness	Pursue research that will benefit individuals, communities, and/or society, and inform stakeholders and environmental decision makers of their findings	Conduct management practices in such a way as to maximize benefits and minimize harms for individuals and their communities
Replacement	Use alternatives to animal models in research	Use simulations or natural experiments when possible	When possible, favor management practices that work indirectly rather than directly on wildlife populations
Reduction	Minimize the number of animals used in research	Minimize sample sizes or keep encounters with wildlife brief	Minimize the number of animals affected by culls and other potentially harmful or disruptive management practices
Refinement	Modify research practices to minimize harm and suffering to animals	Collaborate to minimize impacts on wildlife populations and ecosystems	Minimize the harms of invasive or disruptive management practices on wildlife

The first three values – freedom, fairness, and well-being – are derived from Beauchamp and Childress’ ‘Four Principles of Bioethics’ (1977): autonomy (freedom), justice (fairness), beneficence, and nonmalfeasance (well-being). Developed at the inception of the field of bioethics in the1970s, these principles were introduced as a method for analyzing ethical issues – first in medical ethics, but later in a larger array of bioethical sub-fields. These principles have been influential in the development of ethics policies involving humans.

These three values can be used to analyze the ethical implications of EER with respect to human entities, including local communities, researchers, and conservation managers.^4^ For instance, consideration of ‘freedom’ might inform researcher of the need to avoid jeopardizing locally valuable resources without consultation with, and consent from, local communities whose daily activities might depend on these resources. Consideration of ‘fairness’ might inform researchers to balance the interests of various parties affected by their EER, and consideration of ‘well-being’ might inform researchers of a ‘duty to warn’ or inform stakeholders and environmental decision makers of their findings. While there is overlap between the applications of the values, these three are sufficiently exhaustive to permit articulation of all the ethical implications of EER for human entities.

The last three values – replacement, reduction, and refinement – are derived from the ‘Three Rs of Humane Animal Treatment’ developed by Russell and Burch (1959) to guide the humane use of animals in research. In animal research ethics, ‘replacement’ refers to preference for methods that use nonanimal subjects; ‘reduction’ refers to a preference for methods that use fewer animal subjects; and ‘refinement’ refers to a preference for methods that cause less distress to animal subjects.

These three values can be tailored to apply to a broader range of entities than the nonhuman animals that are direct subjects of observation and experimentation. They can instead be used to analyze the ethical implications of EER with respect to nonhuman biological entities such as individual organisms, species (or other taxonomic groups), populations, communities, ecosystems, and the biosphere. For instance, consideration of ‘replacement’ might direct researchers to use simulations or natural experiments when possible, consideration of ‘reduction’ might guide them to reduce sample sizes or to keep encounters with wildlife brief, and consideration of ‘refinement’ might lead them to collaborate to streamline efforts and maximize the data gathered from a minimal number of samples.

Together, these six values are well suited for ethical analyses of wildlife disease management. Notably, alternative proposals in the literature fail to provide guidance in evaluating the impact of ecological practices on human entities, which is problematic insofar as EER is intimately connected with human objectives (Vucetich and Nelson 2007). We contend that consideration for both human and nonhuman entities must be part of ethical decision making in almost any EER and management context and that this is rendered especially salient in wildlife disease management.^5^

Nowhere than in the subfield of wildlife disease ecology is clearer that human and nonhuman interests are inextricably intertwined; and without providing a framework that puts these interdependencies front-and-center, it will be more difficult to articulate and advocate for management practices that produce mutually beneficial outcomes for humans, wildlife, and conservation (hereafter, referred to as ‘win-win’ outcomes). We hold that, although such beneficial outcomes are not always possible, they are too often overlooked, and that they should serve as the gold standard for wildlife disease management. This is because they are sustainable, and they are consistent with applied evolutionary ecology and ethical conservation.

## Decision tree to aid in ethical reflection on wildlife disease management

To assist in ethical decision making with respect to wildlife disease management, we propose a decision tree. This tree consists of three questions that – in conjunction with the six values listed previously – can guide managers to identify the ethically salient dimensions of the cases they contend with, and to move toward ethically responsible practices in complex situations. It is not intended to serve as an algorithm for obtaining an ethical outcome, but rather as a prompt to help direct one’s attention to the ethically salient parameters that will tend to be relevant in a wide range of different particular cases.

McCallum and Hocking (2005), and later McCallum (2009), identify four conditions under which wildlife diseases raise distinct ethical concerns: when the diseases are zoonotic, when they affect livestock, when they affect endangered species, and when they are deliberately introduced to control pest populations. We take their analysis as our starting point. Notably, we do not explicitly consider cases where pathogens are introduced into wild pest populations as a biological control mechanism in our analysis because these cases (McCallum and Hocking’s final category), insofar as they are relevant to wildlife disease management rather than biological control, are subsumed under the other categories.

The decision tree prompts the identification of a series of ethically salient factors: (i) whether the disease is zoonotic; (ii) whether the disease threatens an endangered species; and (iii) whether the disease threatens a population on which local human communities depend. Each of these factors will guide wildlife disease ecologists to take into consideration a distinct set of ethical questions, articulated through the language provided by the six core values, and with attention to adaptive management and EER. We present the decision tree and then walk through how it would be applied in a real case study.

### Q1: Is the disease zoonotic?

Yes: This indicates that serious intervention might be warranted. Identify who are the vulnerable parties, and in what ways and to what degree are they vulnerable, using the values of fairness, freedom, and well-being.

1. In the case of a potential pandemic (e.g., H1N1, Avian Influenza), the vulnerable parties are many, and they are very vulnerable (the world’s poor, the young, the ill, and the elderly are at highest risk), so strong measures are permissible.
2. In the cases where human infections are few and do not target particularly vulnerable populations (e.g., Bovine Spongiform Encephalopathy/Creutzfeldt–Jakob disease), less interventionist methods might be more appropriate. (Note: this is notwithstanding the economic ramifications for countries dependent on certain export industries, which can be a significant consideration in cases such as this.)
3. In cases where there are few infections, but these target vulnerable populations (e.g., ebola virus contracted from subsistence hunters collecting primate bushmeat in the Republic of Congo), solutions necessarily have to involve finding win-win^6^ outcomes for the local human communities such that they gain value from practices that reduce zoonotic transmission.

In all cases, care should be taken to ensure that disease management practices are minimally harmful to the wildlife and that they conform to the principles of EER.

No: Proceed to Q2.

### Q2: Is the vulnerable population at risk of becoming endangered or extinct?

Yes: Identify the vulnerable parties, and in what ways and to what degree are they vulnerable: how can consideration of replacement, refinement, and reduction inform these practices?

1. Is the species taxonomically rare? Does it perform a vital ecological function (.e.g., keystone species, top predator, etc.,)? Probably some serious intervention is warranted.
2. Species can become endangered because of wildlife disease [e.g., white-nose syndrome and many species of bats (Cryan et al. 2013)] and so a risk assessment associated with the disease and affected populations may be warranted.
3. If not, then perhaps it would be good to think about some less interventionist methods, if available.

In all cases, care should be taken to ensure that disease management practices are minimally harmful to the wildlife and that they conform to the principles of EER.

No: Proceed to Q3.

### Q3: Is the vulnerable population one on which local human communities depend? (Note: ‘local human communities’ is a broad term. It could refer to the entire economy of a country or a small village.)

Yes: To determine whether an intervention is warranted, and to what degree, it is important to identify who are the vulnerable human entities and in what ways and to what degree are they vulnerable, using the values of justice, autonomy, well-being. It is also important to identify the vulnerable nonhuman entities, and the ways in which they are vulnerable, using the values of replacement, reduction, and refinement.

1. If an entire industry were threatened – one with a significant economic consequences for many people (e.g., a mycoplasma outbreak in New Zealand sheep) – strong interventions might be warranted.
2. If the community affected is small and not particularly vulnerable (e.g., local sport hunting would be affected) less interventionist methods might be more appropriate.
3. If the community affected is small, but vulnerable (e.g., toxoplasmosis affecting beluga whales on which an Inuit community is dependent for subsistence), solutions necessarily have to involve finding win-win outcomes for the local human communities such that they gain value from practices that reduce the disease transmission.

In all cases, care should be taken to ensure that disease management practices are minimally harmful to the wildlife and that they conform to the principles of EER.

No: No intervention is likely warranted.

## Case study – showing how the decision tree works

Reconsider the case study introduced earlier: bovine tuberculosis in Eurasian badger populations (Godfray et al. 2013). To see how the decision tree would guide wildlife disease managers in avoiding ethical pitfalls consider the decision tree:

Q1: Although the disease is technically zoonotic (Grange 2001), the risk of zoonotic transmission from badgers is not significant in the UK (de la Rua-Domenech 2006). This removes one indicator that would warrant highly invasive methods.

Q2: Similarly, the disease is not threatening an endangered population. This removes a second indicator.

Q3: The disease does, however, affect cattle – a population highly valued by local human communities. Even if the cattle are sold purely as an export, the economic wellbeing of the local human communities is threatened because their cattle are a vulnerable population.

The vulnerable population is cattle, and the vulnerable parties are not only the cattle but also the farmers whose livelihoods are affected by deaths of cattle, and also other members of the local human community whose businesses might also suffer from a significant loss in the local cattle industry. Vulnerable, also, are the wild badgers that might be subject to violence; they, thus, represent a significant ‘vulnerable party’.

How, then, does this affect the vulnerable human entities in terms of freedom, fairness, and well-being? The freedom of farmers to profit from the goods of their property is clearly threatened. One might also favor measures that result in outcomes that do not unfairly disadvantage these farmers relative to their competitors – for example, where any wildlife disease management procedures that result in increased losses to the farmers is accompanied by adequate financial compensation. Clearly, the wellbeing of the farmers and their communities is undermined by any management practices that increase disease rates in cattle, and even ones that fail to reduce these disease rates. The harm in this case is not to a community of reasonable size, but not significantly vulnerable, insofar as losses to the farmers can be compensated for by the state. This indicates that management practices should only be aggressive if the benefits are clear.

How can we evaluate disease management practices in terms of their impact on nonhuman entities in terms of replacement, reduction, and refinement? Replacement would indicate that managers should opt for methods that only work indirectly rather than directly on the wildlife, such as erecting fences to minimize contact between infected wildlife and domestic livestock, or habitat management including controlled burns to minimize disease risk (Wobeser 2002). Reduction would indicate that, if a more invasive procedure is required, it should affect only a minimal number of animals. Refinement would indicate that, for each animal affected, culls should be a last resort and they should be conducted as humanely as possible.

In the case in question, however, aggressive culling was undertaken without clarity regarding the expected benefits (Independent Expert Panel 2014). Badgers were culled in an inhumane manner (with death times exceeding prescribed limits); large numbers of badgers were affected in addition to the ones culled, as badger social groups were disbanded (Carter et al. 2007). Furthermore, this disbandment caused a spread of the disease, increasing the size of the vulnerable cattle population (Godfray et al. 2013). The end result was significant harms to one vulnerable group – the badgers – without significant benefit to the others – the cattle and the ranchers.

It would seem, then, that aggressive measures were not appropriate in this case, either from an ethical standpoint or from the perspective of EER – at least insofar as one is focused on the third stage of the decision tree. Underlying the management techniques that have been employed to control bovine tuberculosis is the profound evolutionary consequences that result from such management techniques. *M. bovis* has limited genetic diversity on the British Isles, which is consistent with recent bottlenecks and periodic selection (Smith et al. 2006). The consequences for pathogen virulence are unclear, but it is evident that the current pattern of genetic structure and diversity of *M. bovis* populations on the British Isles is the result of roughly a century of disease management practices including culling (Smith et al. 2006).

## Conclusions

This case of bovine tuberculosis in Eurasian badgers clearly exemplifies the need for applied evolutionary ecology and ethical conservation in wildlife disease management practices. It also indicates how the procedure proposed in this study – the decision tree informed by six central values – can assist in this undertaking. It is important to note that, regardless of which branches of the tree are invoked, in all cases wildlife disease management practices must be undertaken in a manner consistent with EER. This means both (a) being mindful of the long-term evolutionary and ecological consequences of any disease management strategy employed, not just short-term crisis management, and (b) collecting data such that future disease management practices are improved. Note that (b) is also consistent with engaging in disease management practices that contribute to the well-being of the disease carrying wild population, which will make even invasive or harmful management practices more ethical.

Effective wildlife disease management *is* ethical wildlife disease management, and vice versa. This necessarily involves identifying the vulnerable parties, and devoting problem-solving energy toward finding means by which the management techniques can best serve them. It also involves prioritizing sustainable methods and knowledge development over crisis management or political expediency. To accomplish this, applied evolutionary ecologists have to be integral to the decision making – not only to identify futile versus fertile efforts, but also to ensure whatever measures are taken result in the development of scientific knowledge about the populations that are managed. This will promote management practices with minimal negative impact on wildlife and maximal gain for all vulnerable populations and parties.

## Footnotes

1 Throughout the manuscript, we use the term ‘pathogen’ to refer to any relevant disease or disease vector, including pathogen or parasite.

2 It is beyond the scope of this study to defend the authors’ meta-ethical background assumptions; however, the reader might benefit from knowing that the reasoning herein is informed by a pluralist and naturalized approach to ethics. On this view, ethical rules are tools that we use to navigate the social contexts in which we evolved, both biologically and culturally; and while some rules are better than others in many contexts, no single such tool (such as utilitarian calculus or the Golden Rule) is better than all others across all contexts. This position is not standard in the philosophical tradition, but it is gaining ground and, more importantly, it is the most defensible one, in our view. Nevertheless, the arguments presented herein should be compelling to readers who subscribe to neither a naturalized ethics nor a pluralized one.

3 For more detail on how this might be put into practice, see Paquet and Darimont (2010) and Wall (2010).

4 Our interpretation of these first three values is open to the possibility that they might apply not only to humans and human communities, but also to nonhuman animals and their communities when these beings manifest complex social, cognitive, and affective characteristics. These values might be applicable, for example, to great apes and their communities.

5 Some might object to our distinction between values that apply to human versus nonhuman entities, contending that it evinces a problematic acceptance of ‘human exceptionalism’ – a commitment responsible for the devastation humans have imposed on ecosystems across the planet. Human exceptionalism, however, has multiple interpretations, not all of which are unjustified. To the extent that humans are responsible for said environmental devastation, one form of human exceptionalism would assign special responsibility to our species for remediating these effects. We take this to be uncontroversial. Additionally, however, insofar as humans are cognitively sophisticated social animals, we have developed social systems that include special responsibilities to our conspecifics and that do not always clearly apply to other species. As such, an elaboration of the responsibilities we hold to human and nonhuman entities will need to draw on different conceptual tools (here, core values), not necessarily because these human and nonhuman entities are radically different in kind, but rather because of constraints imposed by the sociocultural history in which those tools were developed. Indeed, we are open to the proposal that parallel social contracts exist within some nonhuman communities, and that social contracts exist between humans and some nonhumans (dogs being the clearest potential example); but the articulation of an ethical framework sufficient to clarify these distinctions (though of considerable interest to the authors) lies beyond the scope of this study.

6 By ‘win-win’ we refer to outcomes that are mutually beneficial to not only human communities, but also to the animals, populations, and ecosystems that they occupy.
